# Potential of Ex Situ Conservation Strains Revealed by Genetic Analysis of Oceanic Islands' Endangered Species *Pittosporum parvifolium*

**DOI:** 10.1002/ece3.70506

**Published:** 2024-10-30

**Authors:** Haruna Kawakita, Shota Sakaguchi, Saeko Katoh, Hidetoshi Kato, Takefumi Tanaka, Yoshiteru Komaki, Takahito Ideno, Hiroaki Setoguchi

**Affiliations:** ^1^ Graduate School of Human and Environmental Studies Kyoto University Kyoto Japan; ^2^ Department of Biological Science, Graduate Schools of Science and Engineering Tokyo Metropolitan University Tokyo Japan; ^3^ Botanical Gardens, Graduate School of Science The University of Tokyo Tokyo Japan

**Keywords:** critically endangered species, genetic diversity, genetic purity, insular endemic, microsatellite

## Abstract

The Ogasawara Islands, representing an oceanic island ecosystem in Japan, have a notably high rate of endemic species akin to other oceanic islands globally. *Pittosporum parvifolium* is a critically endangered shrub with only four remaining individuals in its natural habitat on the Ogasawara Islands. Current conservation efforts encompass both in situ and ex situ approaches for *P. parvifolium*. However, these efforts face challenges stemming from the lack of critical conservation information. Therefore, we explored *P. parvifolium*'s genetic diversity and implications for conservation. We utilized simple sequence repeat markers to scrutinize genetic diversity within both in situ and ex situ populations, revealing notably rich diversity among both. The in situ genetic diversity was significantly high despite the few extant individuals. In addition, many of the ex situ peculiar genotypes were absent in individuals conserved in situ. This investigation also provides insights into the reproductive strategies and combinations of selfing and outcrossing. The results of the present study recommend conservation to maximize genetic diversity in *P. parvifolium* by promoting cross‐pollination among in situ individuals and by introducing individuals with unique genotypes into ex situ stocks.

## Graphical abstract

The Ogasawara Islands, home to the critically endangered shrub *Pittosporum parvifolium*, have seen significant conservation efforts, yet challenges remain due to insufficient critical information. Our study used simple sequence repeat markers to reveal high genetic diversity in both in situ and ex situ populations, with unique genotypes found in each. We recommend enhancing conservation strategies by encouraging cross‐pollination among in situ individuals and integrating individuals with unique genotypes into ex situ conservation efforts to maximize genetic diversity.

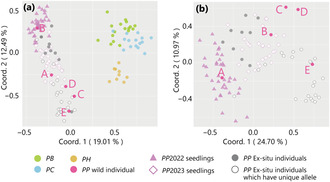

## 1 Introduction

Numerous wild plant species are currently classified as rare or endangered and face threats across diverse global habitats. In Japan, of the approximately 7000 vascular plant species, 1790 are designated as endangered according to the International Union for Conservation of Nature (IUCN) criteria (Ministry Environment of Japan 2020). Conservation efforts for these imperiled species involve safeguarding and fostering populations within and outside their native environments. The foremost strategy to prevent plant extinction is in situ conservation, but this is not always feasible in many areas (Havens et al. 2006; Paparella et al. 2015). In such scenarios, ex situ conservation serves as a contingency for species that are vulnerable or lost in their natural settings (Li and Pritchard 2009). Botanical gardens and similar facilities play a crucial role in nurturing endangered plant species and preserving seeds, protecting approximately 30% of all species globally and approximately 41% of known threatened plant species (Mounce, Smith, and Brockington 2017). Ex situ conservation efforts conducted in botanical gardens are pivotal for the protection and management of endangered plant species, supporting both in situ habitat management and species‐level conservation efforts (Havens et al. 2006). Genetic diversity and population fitness are essential in conservation practices to ensure the long‐term viability of a species (Friar et al. 2000; Robichaux, Friar, and Mount 1997).

An oceanic island emerges through the uplift of submarine volcanoes and is characterized by its historical isolation from any continent (e.g., Whittaker and Fernández‐Palacios 2007). The biological colonization of such islands relies heavily on species introduced from continents, thereby constraining the diversity of species that can successfully inhabit these islands. Consequently, a distinct biological community tends to evolve, marked by the absence of key continental species (e.g., oaks and conifers) and the presence of unique island‐adapted species (Carlquist 1974). However, the intrinsic value of such ecosystems is affected by their susceptibility to anthropogenic disturbances (Kato et al. 1999; Stuessy 2020), including the introduction of highly invasive alien species through human activities. The Ogasawara Islands, representing an oceanic island ecosystem in Japan, epitomize this pattern, with a notably high rate of endemic species akin to other oceanic islands globally. Alarmingly, approximately 82.3% of the 124 species endemic to the Ogasawara Islands, totaling 102 species, are classified as endangered (Toyoda 2014).

This study specifically targeted the driest and lowest tree‐height forests, referred to as “sclerophyllous shrublands,” a type of vegetation characterized by hard, leathery, evergreen foliage that is specially adapted to prevent moisture loss, such as Mediterranean vegetation (UNESCO: https://whc.unesco.org/en/list/1362) (Figure S1a). Notably, this vegetation harbors several rare and endangered shrub and/or tree species, including *Callicarpa nishimurae*, *Symplocos kawakamii*, and *Myrsine okabeana* (Shimizu and Tabata 1991), as well as numerous other endangered species native to environments susceptible to artificial disturbances, such as human activity (including past landuse and construction of tourism facilities) and foraging damage inflicted by feral goats (Shimizu 2003). This highlights the urgent necessity for comprehensive conservation strategies to safeguard the fragile ecosystems of the Ogasawara Islands.

**FIGURE 1:**
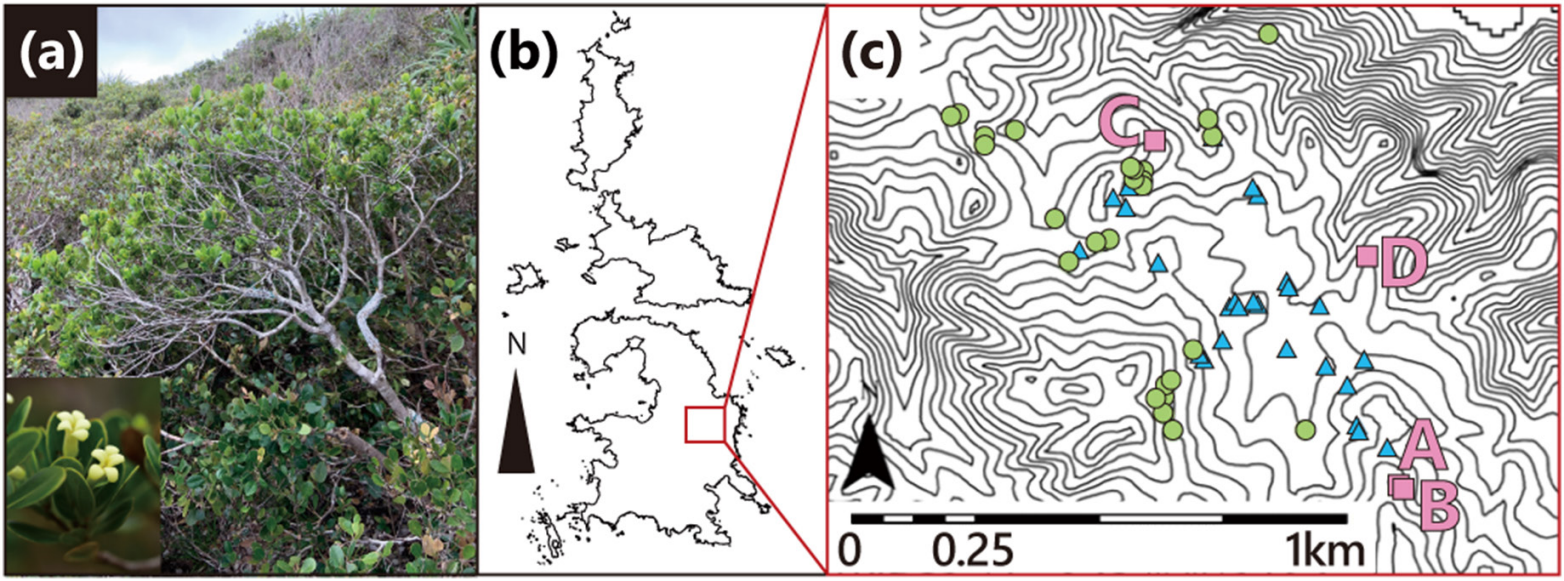
Morphological characteristics and habitat of *Pittosporum parvifolium*. (a) Tree and flower morphology of *P. parvifolium*. (b) The Chichijima Archipelago in the Ogasawara Islands, Japan. (c) Distribution of individuals of each of the three parapatric species on the eastern slope in Chichijima Island: Pink squares, green circles, and blue triangles represent *P. parvifolium*, *P. boninense,* and *P. chichijimense*, respectively. Additionally, the pink markers labeled A, B, C, and D represent the wild *P. parvifolium* individuals, currently surviving in their natural habitat.

*Pittosporum parvifolium* Hayata (Figure 1a) is one such species. It is a woody plant belonging to the genus *Pittosporum* (Pittosporaceae) and is endemic to the Chichijima Archipelago (Figure 1b) within the Ogasawara Islands (Toma 2017). “Adaptive radiation” is a phenomenon in which a single ancestor rapidly differentiates into three or more endemic species. It is an important phenomenon in speciation studies on oceanic islands, which are considered experimental sites for evolution. Four species of the genus *Pittosporum* are endemic to the Ogasawara Islands: *P. parvifolium*, *P. boninense*, *P. chichijimense*, and *P. beecheyi*. These species are considered to have experienced adaptive radiation. It has been reported that the four species likely evolved from a single ancestor (Kawakita and Setoguchi 2020). The reason why this species deserves conservation is not only its rarity but also that it is an important species in terms of evolutionary genetics.

As of 2023, only four wild individuals have been confirmed to persist on Chichijima Island (Figure 1b), with no observed natural regeneration, rendering its conservation status highly critical. The ecosystem inhabited by *P. parvifolium* reportedly experienced significant disturbances on two occasions: During the Meiji period (approximately 150 years ago) with the onset of settlement and again following World War II (Shimizu 2003). Subsequently, with the establishment of tourist facilities such as observation decks, walking trails, and gardens before it was designated National Park in 1974, only seven individuals were found in their natural habitat. Three of the seven individuals withered, and only four individuals are currently surviving after World Heritage registration (Toyoda 2003) and grant of World Heritage Site status in 2010. In addition, cuttings from one of the three withered individuals are surviving in the botanical garden of the University of Tokyo and have been cultivated for ex situ conservation of the withered wild individuals. Considering the origin and unique genotype of the cutting individuals, we designated them as “wild individuals” in this study (see Table 1). Current conservation efforts encompass both in situ and ex situ approaches for *P. parvifolium*. In the habitat area (in situ) where existing individuals thrive, protective measures such as fence cages have been implemented to shield them from the detrimental effects of invasive species, such as feral goats and black rats, which are recognized as significant contributors to mortality (Figure S1b). These pests often inflict damage by gnawing on the trunks, leaves, and fruits of native plants, with individual tree vigor notably declining because of trunk gnawing near the root (Kawakita, personal observation). These individuals are believed to be affected by the abovementioned harmful factors influences as well as by drought, and three individuals that once thrived in the area have died.

**TABLE 1:** Population genetic measures were given for in situ and ex situ *Pittosporum parvifolium* and other endemic *Pittosporum* species.

Species	Status	*N*	*N*_A_	*A*_R_	*n*_e_	*P*_A_	*H*_O_	*H*_E_	*F*_IS_
*P. parvifolium*	Wild	5	3.92 (0.66)	4.33 (0.62)	3.06 (0.60)	1	0.60 (0.09)	0.56 (0.06)	−0.07
	2022 seedlings	37	2.08 (0.23)	2.38 (0.23)	1.85 (0.12)	2	0.46 (0.06)	0.42 (0.06)	−0.10
	2023seedlings	30	2.75 (0.22)	3.17 (0.26)	2.43 (0.23)	0	0.66 (0.07)	0.55 (0.04)	−0.19
	Ex situ	31	4.00 (0.73)	3.60 (0.42)	2.90 (0.49)	3	0.57 (0.09)	0.55 (0.06)	−0.03
*P. boninense*	Wild	17	8.10 (1.87)	4.79 (0.59)	5.26 (1.64)	38	0.54 (0.07)	0.63 (0.07)	0.15*
*P. chichijimense*	Wild	15	8.75 (1.49)	5.24 (0.60)	5.31 (1.37)	47	0.49 (0.08)	0.65 (0.07)	0.24*
*P. beechey*	Wild	9	3.25 (0.35)	3.31 (0.25)	2.27 (0.24)	16	0.40 (0.08)	0.49 (0.07)	0.19*

*Note:* Standard errors are given in brackets.

Abbreviations: *N* = number of individuals, *N*_A_ = number of different alleles, *A*_R_ = allelic richness, *n*_e_ = number of effective alleles, *P*_A_ = number of private alleles, *H*_O_ = observed heterozygosity, *H*_E_ = expected heterozygosity, *F*_IS_ = fixation index.

* Significant deviation of *F*_IS_ from zero (*p* < 0.01).

Simultaneously, an ex situ conservation initiative is underway at the Koishikawa Botanical Garden, University of Tokyo. A variety of approaches have been employed, including the cultivation of cuttings from native plants, the cultivation of seeds sourced from native plants, and the cultivation of seeds obtained from individuals conserved ex situ. However, these efforts face challenges stemming from the lack of critical conservation information. For instance, in an in situ setting, the extent of hybridization within natural habitats remains unknown, and the origins of naturally fruiting seeds have yet to be determined. Similarly, ex situ genetic relationships with certain wild individuals remain unknown, as is the connection with related species within the garden's confines.

Previous studies that have examined the genetic diversity of endangered species on the Ogasawara Islands are reviewed below. *Stachyrus macrocarpus* var. *prunifolius* is as endangered as *P. parvifolium*, and conservation activities have been promoted by genetic analysis. This woody species is exclusive to the Hahajima Island within the Ogasawara Archipelago. Genetic analysis employing simple sequence repeat (SSR) markers on 300 seedlings propagated from seeds sourced from three wild individuals and their progeny revealed that alleles were absent in the known parental specimens. This advancement has facilitated the identification of previously undetected wild individuals within their natural habitats (Kaneko, Abe, and Isagi 2013), illustrating the pivotal role of genetic analysis in conservation efforts. In contrast, *Rhododendron boninense*, like *P. parvifolium*, has experienced a significant decrease in its wild population, resulting in minimal genetic diversity. Isagi et al. (2020) reported the observed and expected heterozygosity values of 0.0 for this species. This low diversity can be partly attributed to repeated generational transitions within a small population and the accumulation of deleterious variations. Previous studies have suggested that the genetic diversity of *P. parvifolium* (observed heterozygosity: 0.31; expected heterozygosity: 0.30) (Katoh et al. 2013) is similar to that of *S. macrocarpus* var. *prunifolius* (observed heterozygosity, 0.62; expected heterozygosity, 0.48) (Kaneko, Abe, and Isagi 2013). This is thought to be due to the dioecious nature of genus *Pittosporum*. However, because the population size remains small, genetic management interventions are essential to mitigate future inbreeding and bottleneck effects. This is particularly important for species such as *P. parvifolium*, for which recovery through the implementation of appropriate conservation measures is expected.

It is crucial to adopt insights into in situ and ex situ conservation to protect endangered species (e.g., reintroducing rare, unique alleles in ex situ to in situ populations by artificial pollination and seed introduction), maximize genetic diversity, and increase the number of individuals. In addition, genetic information can be used to select pure *P. parvifolium* ex situ cultivation stocks and in situ next‐generation individuals for further conservation projects.

The objective of this study was to contribute to the conservation of the critically endangered species *P. parvifolium* by harboring and/or enhancing genetic diversity in both natural habitat populations and ex situ conservation populations located in botanical gardens. First, we propagated seedlings originating from seeds of extant individuals in natural habitats for two seasons (2022 and 2023) for in situ conservation. Second, 12 microsatellite loci were used for detecting the genetic diversity of the “extant wild individuals,” “propagated seedlings in 2022” and “2023” as in situ conservation groups, and “all cultivation stocks in botanical gardens” as ex situ conservation groups. Finally, investigations were undertaken to explore the potential for hybridization with related species that parapatrically inhabit narrow areas (see Figure 1c).

## 2 Materials and Methods

### 2.1 Study Sites and Phylogenetic Relationship With Another Ogasawara Endemic *Pittosporum* Species

The study site is located on the Chichijima Island in the Ogasawara Islands (Figure 1b) in the northwestern Pacific, approximately 1000 km south of the Japanese mainland. The Ogasawara Islands are characterized as subtropical oceanic islands (26°30′ to 27°40′N and 142°00′ to 142°15′ E) derived from volcanic activity (Ono 1991). In 2011, these islands were designated as UNESCO World Heritage Sites because of their remarkable levels of endemism in both land snails and vascular plants and the unique components of plant species of sclerophyllous shrublands confined to the northeastern coast of the Chichijima and Anijima Islands (both islands are part of Chichijima Archipelago). The distinction between the Ogasawara Islands lies in their unique combination of concentrated endemism and extensive adaptive radiation, setting them apart as exemplars of evolutionary processes (UNESCO 2011).

*Pittosporum parvifolium* (Figure 1a), which is also one of the evergreen organization species, has a tree height of up to 0.5–1.5 m (Toyoda 2014). Further, *P. parvifolium* is not only important for conservation because of its rarity but also for evolution because it is included in the group considered to be an adaptive radiation that is actively studied in oceanic islands. The four endemic species of the genus *Pittosporum* found on the Ogasawara Islands constitute a monophyletic group, with their closest relatives identified as *P. tobira* and *P. illicioides*, which are indigenous to Japan and China, respectively (Kawakita and Setoguchi 2020). All four endemic species are diploid with a chromosome count of 2*n* = 24 (Ono and Masuda 1981). Among these species, *P. beecheyi* exclusively inhabits the Hahajima archipelago, whereas *P. boninense* is found in both the Hahajima and Chichijima archipelagos. However, both *P. parvifolium* and *P. chichijimense* are confined to the Chichijima Archipelago. Despite these differences in natural habitats, the botanical garden cultivates all *Pittosporum* species in pots at one place. Figure 1c illustrates the distribution of the three species in close proximity to the Chichijima Island.

### 2.2 Sampling for In Situ and Ex Situ Genetic Assessment

Seedling samples utilized for the in situ genetic assessment were cultivated by collecting seeds from wild individuals (Figure S1c) and subsequently sowing and nurturing the seedlings within their natural habitat. The decision to raise seedlings from seeds stemmed from the convenience of identifying the pollen parent (father) when the seed parent (mother) was known (mother tree, i.e., seed‐collected individuals, has been recorded for most of the seedling cultivars in botanical garden). The plants yielding fruit during the study were assigned management numbers “B” in 2021 and “A” in 2022 (Figure 1c), with both years' seeds resulting from natural fertilization. The total number of obtained seedlings was as follows: 37 seedlings collected in December 2020 and cultivated from December 2020 to July 2022, labeled as “2022 seedlings,” and 30 seedlings collected in December 2022 and grown from December 2022 to June 2023, termed “2023 seedlings” based on their crop year. One leaf was collected from each seedling. Additionally, DNA was extracted from four extant wild *P. parvifolium* individuals, denoted as A, B, C, and D, and assigned the respective management numbers (Figure 1c), serving as potential paternal parents.

For ex situ verification, leaf samples were collected from each of the 38 pots housing *Pittosporum parvifolium* specimens maintained at the Koishikawa Botanical Garden, University of Tokyo (Figure S1d). Among these 38 plants, five were identified as cuttings from native individuals based on the information provided on cultivation management tags. As a potential progenitor of the extraterritorial conservation stock, it is possible that a native individual, which is currently nonexistent, could have served as the parent. Hence, alongside the four existing individuals, cutting cultivars originated from withered wild individuals at the Koishikawa Botanical Garden were also assigned to candidate parents. Therefore, we included a fifth individual, possessing candidate parent genetic material from this ex situ conservation population.

Subsequent samples were included to ensure a comprehensive analysis and verify the presence or absence of hybridization in the next generation of individuals within the botanical garden. In particular, three closely related species (*P. boninense*, *P. chichijimense*, and *P. beecheyi*) are cultivated in close proximity to where *P. parvifolium* is found in botanical gardens, and it is imperative to investigate the potential for hybridization in the next generation of individuals within the garden. However, due to the limited number of pots containing these closely related species in the garden (ranging from one to three), which did not provide a sufficient sample size for genetic analysis, additional samples were sourced from wild individuals to augment the sample set. Specifically, 17 samples were collected from *P. boninense*, 15 from *P. chichijimense*, and 9 from *P. beecheyi*.

### 2.3 DNA Extraction and Fragment Analysis Using Simple Sequence Repeat (SSR)

All collected samples were dried and stored in silica gel. Notably, leaf samples from seedlings were obtained in smaller quantities (approximately 0.006–0.008 g) than those collected from mature trees. Each sample was then placed in a 1.5‐mL tube containing a stainless‐steel grinding bead and pulverized using a grinder. The resulting powder was treated with a polysaccharide removal solution in HEPES buffer (pH 8.0; Setoguchi and Ohba 1995) and washed twice. Subsequently, DNA extraction was carried out using the CTAB method (Doyle and Doyle 1987), with the extracted DNA dissolved in 20 μL of TE buffer and stored for subsequent analysis.

In accordance with Katoh et al. (2013), SSR markers at 12 loci were employed for genetic analysis and were selected based on their demonstrated efficacy in detecting genetic polymorphisms (Table S1). These markers were chosen to ensure a robust and comprehensive genetic characterization of the samples under study.

For the PCR reaction, 9 μL of premix was first made per sample: 4.72 μL of 2X Multiplex PCR Buffer (Multiplex PCR Assay Kit ver. 2, Takara Bio, Kusatsu, Japan), 0.05 μL of Multiplex PCR enzyme mix (Multiplex PCR Assay Kit Ver.2, Takara Bio, Kusatsu, Japan), forward (5 pmol), and reverse (5 pmol) primers in 4.23 μL. This premix was dispensed at 9 μL for 1.2 μL of DNA in a sample, and a total volume of 10 μL was set up for PCR. The PCR reaction consisted of an initial thermal denaturation at 94°C 1 min 1 cycle, followed by a secondary thermal denaturation at 94°C 30 s, primer annealing at 60°C 1 min, and extension at 72°C 1 min for all primer pairs, followed by 30 cycles of these three steps and a final extension at 72°C 10 min 1 cycle. The samples were analyzed using an ABI Prism 3130 Genetic Analyzer (Applied Biosystems) with a GeneScan 600 LIZ size standard, POP7 polymer, and a 36‐cm capillary array (Applied Biosystems). The DNA fragment sizes were scored by the same person using GENEMAPPER 3.7 (Applied Biosystems).

### 2.4 Using Software for Genetic Analysis

This study utilized a variety of analytical techniques to investigate the genetic relationships, clonality, paternity, and population dynamics in both in situ and ex situ populations of *P. parvifolium*. Principal coordinate analysis (PCoA) was performed using GenAlEx 6.5 (Peakall and Smouse 2012) to assess hybridization with related species in both in situ and ex situ populations. The results were visualized using the “ggplot2” library (Wickham 2009) in R ver. 4.1.2. Clonal identification among the 38 ex situ individuals was conducted using GenAlEx 6.5. Paternity analysis was carried out using Cervus v.3.0 software (Marshall et al. 1998; Kalinowski, Taper, and Marshall 2007) across 12 loci. On the Ogasawara Islands, *P. parvifolium* exhibits a shift in its sexual expression, being predominantly dioecious but occasionally a hermaphrodite, as documented by Toma (2017). Consequently, in paternity analysis, verification under the assumption of self‐fertilization was conducted. Population‐level genetic diversity (*H*_o_, *H*_e_, and *F*_IS_) was assessed using GenAlEx 6.5, while allelic richness was calculated using the R package “hierfstat” (Goudet 2005). Deviations of the *F*_IS_ from the Hardy–Weinberg equilibrium were verified using the online tool Genepop ver. 4.7 (Raymond and Rousset 1995). The significance of genotypic disequilibrium for each population was also tested with probability tests by Genepop. Individual level heterozygosity was also estimated using GenAlEx 6.5, and significant differences were examined by a Tukey's HSD multiple comparison using the R package “multcomp”. The effective population size was estimated using the software NeEstimator2 (Do et al. 2014), employing the molecular coancestry method. These analyses provided comprehensive insights into the genetic structure, diversity, reproductive dynamics, and effective population size of *P. parvifolium* populations, both in their natural habitat and botanical garden settings.

## 3 Results

### 3.1 Samples Used for Genetic Analyses

Prior to genetic analyses of *P. parvifolium*, we identified clonal individuals to avoid duplication of identical genotypes attributed to cutting propagation in botanical gardens as ex situ conservation. Using GenAlEx 6.5, identical genotypes were selected as potential clones, and cultivar management ledgers and tags in the pots were checked. As a result, six samples were excluded from a total of 38 samples, leaving 31 samples for ex situ analysis, while one sample, which had died and was no longer growing in situ, was used as a candidate parent for further genetic analysis, i.e. samples (*n* = 5), including the ex situ conservation stock E, with a wild genotype, were established as the parental genotype for genetic analysis of the ex situ conservation stock. In addition, 37 and 30 seedlings sewn in 2022 and 2023, respectively, were also included in the genetic analyses (Tables 1 and 2). These seedlings originated from natural pollination for in situ conservation.

**TABLE 2:** Estimated effective population size of *Pittosporum parvifolium*.

Population	Ne	Confidence interval (CI)
Wild	27.8	0.0–139.4
Ex situ	6.6	3.0–11.6
2022 seedlings	7.4	3.4–12.9
2023seedlings	3.9	2.5–5.6

*Note:* Effective population size (Ne) was estimated by NeEstimator2. We adopted its molecular coancestry method results with 95% confidence intervals.

### 3.2 Genetic Diversity

#### 3.2.1 Interspecies Comparison

The numbers of different alleles across the 12 loci in wild *P. parvifolium*, *P. boninense*, *P. chichijimense*, and *P. beecheyi* were 5, 17, 15, and 9, respectively (Table 1). Population genetic diversity was consistently high across all four species, with allelic richness (A_R_) ranging from 5.24 (*P. parvifolium*) to 3.31 (*P. beecheyi*), with *H*_E_ ranging from 0.65 (*P. chichijimense*) to 0.49 (*P. beecheyi*). The fixation index (*F*_IS_) was approximately zero in *P. parvifolium*. In contrast, the values in other endemics range from 0.15 (*P. boninense*) to 0.24 (*P. chichijimense*), which can be significantly deviated from zero (*p* < 0.001). Thus, wild *P. parvifolium* obeys Hardy–Weinberg equilibrium (HWE), whereas other endemic species harbor excess homozygosity (Table 1).

#### 3.2.2 Genetic Signature in In Situ Seed Propagations and Botanical Garden's Stocks as Ex Situ Conservation in *P. parvifolium*

**FIGURE 2:**
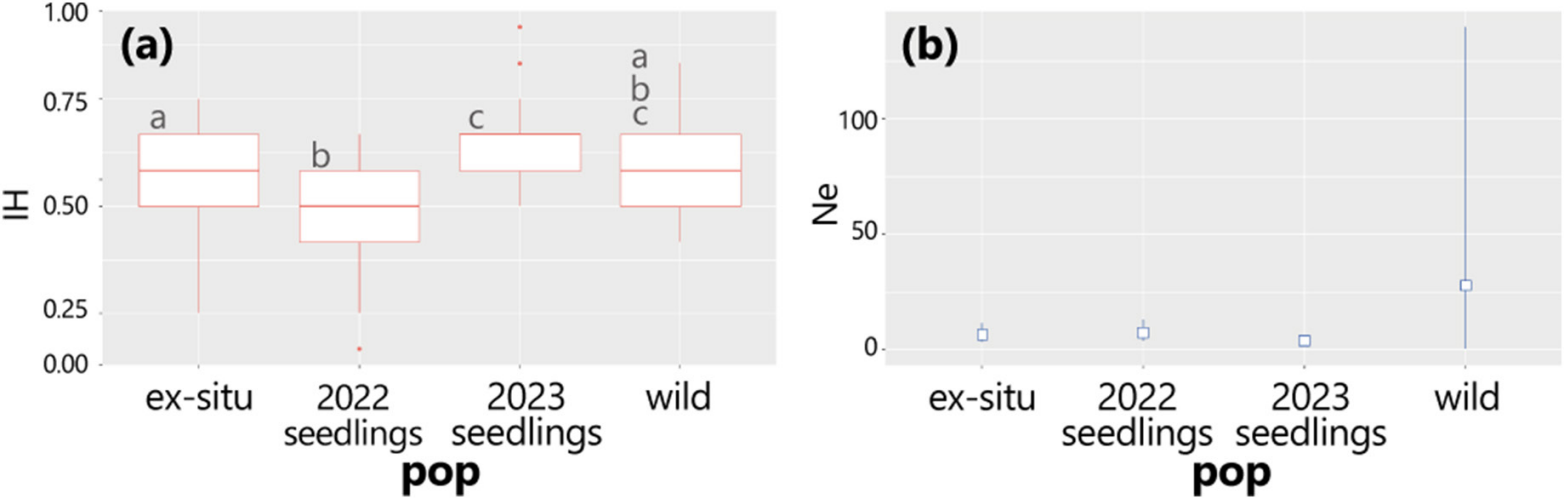
Box plot of the individual heterozygosity (IH) and size range of the estimated effective population size (Ne). (a) The IH depicted in red, and (b) the Ne in the right panel, shown in blue, for four populations of *Pittosporum parvifolium*. The letters on the left shoulder of the boxplots indicate significant differences between the populations (*p* < 0.05).

Thirty‐seven (the seedlings name is called “2022 seedlings”) and thirty (it is called 2023seedlings) samples (Table 1) that originated from natural mating were subjected to SSR analyses. The observed heterozygosity (*H*_O_) ranged from 0.46 (2022 seedlings) and 0.66 (2023seedlings) (the average of both seedling sets was 0.57), while the expected heterozygosity (*H*_E_) varied from 0.42 (2022 seedlings) to 0.55 (2023seedlings) averaging at 0.52. *F*_IS_ was approximately zero (−0.10 in 2022 and −0.19 in 2023seedlings) with no significant deviation from zero, according to HWE. A multiple comparison test revealed significant differences between the 2022 and 2023seedlings from the ex situ conserved plants. In contrast, the wild population did not exhibit significant differences from the other three populations (Figure 2a). The ex situ conservation population, comprising 31 cultivars in botanical gardens, harbored genetic diversity with *A*_R_ = 3.6, *H*_O_ = 0.57, *H*_E_ = 0.55, and *F*_IS_ = ‐0.03 (Table 1), suggesting high genetic diversity within the HWE population, which is comparable to wild individuals.

**FIGURE 3:**
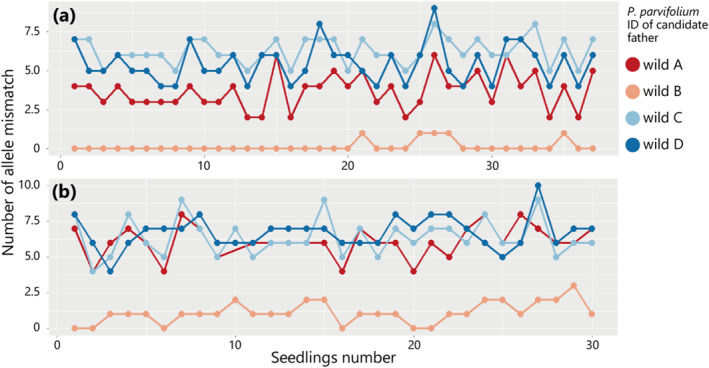
Results of allele mismatch through brute force paternity analysis across all seedlings. (a) Result of 2022 seedlings (*N* = 37) derived from wild B individual as a maternal tree. (b) Result of 2023 seedlings (*N* = 30) derived from wild A as a maternal tree.

Paternity analysis was conducted on 2022 seedlings (wild B as the seed parent), using four wild individuals (wild A, B, C, and D) as potential fathers. The results revealed that all seedlings resulted from selfing by wild B (Figure 3a). Similarly, 2023seedlings (wild A as the seed parent) underwent the same analysis, and all seedlings in the second year were found to result from outcrossing with wild B as the father (Figure 3b). The average number of mismatches among the 2022 seedlings was 0.14 when wild B was their father, while for the 2023seedlings, the average number of mismatches was 1.07 when wild B was also their father. Notably, allele mismatches that did not reach zero in some cases could have been due to genotyping errors or branch‐specific mutations.

Estimation of the effective population size (Ne) for the four populations of *P. parvifolium* revealed intriguing findings (Figure 2b). Values larger than the actual population size (*N*) were obtained for the wild *P. parvifolium* population despite the remarkably small number of individuals (Ne = 27.8, *N* = 5). Conversely, the value of the Ne of seedlings derived from selfing wild B (2022 seedlings) or from the crossbreeding between wild A and B (2023seedlings) was smaller than the number of seedlings (Ne = 7.4 and 3.9 for 2022 seedlings with *N* = 37 and 2023seedlings with *N* = 30, respectively). Furthermore, the Ne of *P. parvifolium* under ex situ conservation was low despite the large number of individuals included (Ne = 3.9, *N* = 3.1).

### 3.3 Genetic Cohesion of *P. parvifolium*

**FIGURE 4:**
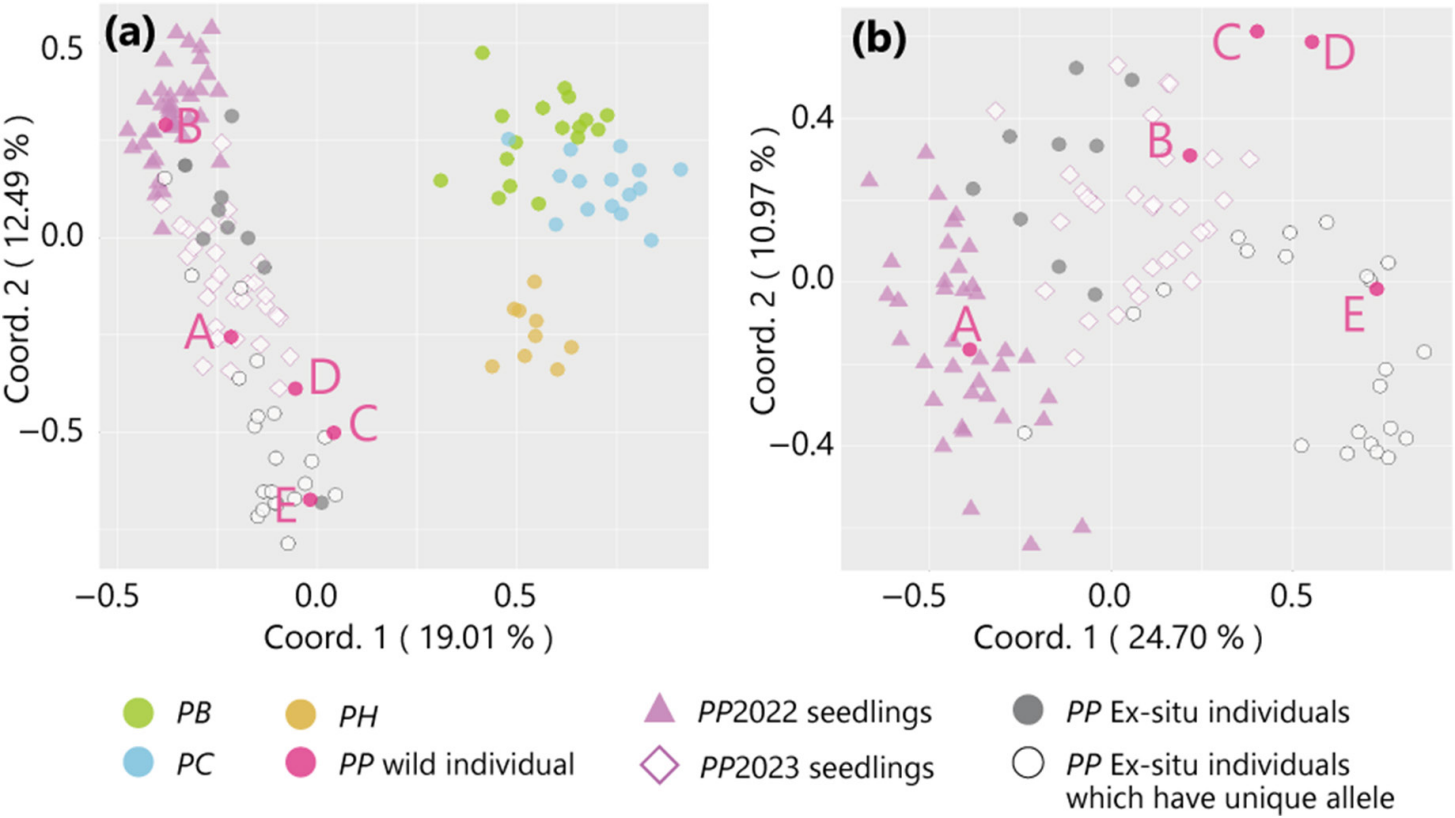
Results of the principal coordinate analysis (PCoA) based on SSR genetic markers. (a) All samples. (b) All individuals of *Pittosporum parvifolium* in in situ and ex situ conservation. PB, *P. boninense*; PC, *P. chichijimense*; PH, *P. beecheyi*, and PP, *P. parvifolium*.

The PCoA results for the in situ analysis revealed distinct clustering patterns. The group consisting of existing *P. parvifolium* individuals (A, B, C, and D), along with the 2022 and 2023seedlings, formed a tight cluster separate from the groups containing *P. boninense* (PB) and *P. chichijimense* (PC), which are related species that grow parapatrically (Figure 4a). Notably, the 2022 seedlings obtained from wild individual B were closely clustered with PCoA dots around wild B, while the 2023seedlings obtained from wild individual A were concentrated between the clusters of wild individuals A and B. These findings strongly suggest that *P. parvifolium* and its in situ seedlings are genetically pure *P. parvifolium* individuals that exhibit distinct genetic separation from related species.

### 3.4 Ex Situ Stocks' Species Cohesion and Genetic Status

A PCoA was performed on the ex situ individuals, showing their clustering with the *P. parvifolium* population and distinguishing them from three genetically related species displayed alongside them in the botanical garden, grouped with the *P. beecheyi* group and the *P. boninense* and *P. chichijimense* groups (Figure 4a). These findings strongly suggest that ex situ conserved individuals of *P. parvifolium* maintain their genetic purity as distinct species.

Notably, during our analysis, we discovered that the ex situ individuals harbored unique alleles at 2 of the 12 loci, which were absent in both wild *P. parvifolium* individuals and the three closely related species (Figure 4a,b, indicated by hollow circle). In Table S2, samples labeled A to E represent parental individuals with the wild *P. parvifolium* genotype, while samples labeled with a “*k*” prefix in the last two rows denote the ex situ individuals. Notably, these ex situ individuals possessed three unique alleles, which are denoted by asterisks. Individuals carrying these distinctive alleles are depicted as hollow circle in the PCoA diagram, as observed in a previous study. Notably, they appeared to be relatively closer to individual E than to the extant wild individuals (A–D) or were slightly positioned further away from any individual (Figure 4b).

## 4 Discussion

### 4.1 Genetic Integrity of *P. parvifolium* Among Parapatric Three *Pittosporum* Species

Although closely related species grew in parapatry, mature wild trees and naturally fruiting seedlings were identified as genetically pure *P. parvifolium* (Figure 4). The genetic purity of *P. parvifolium* could be attributed to the ecological characteristics of the endemic species on Chichijima Island, that differed from others, with respect to (1) habitat preference and (2) flowering periods.

The habitat preferences of the three parapatric species are highly differentiated, as described in the Introduction section: *P. parvifolium* inhabits sclerophyllous shrublands that are characterized by hard, leathery, evergreen foliage vegetation in a dry environment (UNESCO 2011), whereas *P. chichijimense* inhabits the understory within the mesic forest. *P. boninense* is a canopy species within the open and/or edges of forests with sufficient sunlight. These three habitat preference differences can be a strong barrier to gene flow among species, including the prezygotic isolation of pollination/seedling selection. These heterogeneous environments are typical phenomena at the oceanic islands. Additionally, adaptive radiation of plant species is attributed to the heterogeneous factor (Fernández‐Palacios and de Nicolás 1995): *Metrosideros polymorpha* in the Hawaii Islands (Choi et al. 2021), and *Elaeocarpus photiniifolia* in the Ogasawara Islands (Sugai et al. 2013) are two examples of species demonstrating phenotypic changes in response to heterogeneous environments factor on oceanic islands.

Flowering periods play a crucial role in reproductive isolation (e.g., prezygotic isolation) among related species (Coyne and Orr 1997, 2004). The endemic species of *Pittosporum* on Chichijima Island, namely *P. chichijimense*, *P. boninense,* and *P. parvifolium*, exhibit specific flowering periods of March–April, April–May, and April–November, respectively (Toyoda 2003). However, while the number of seeds from spring‐flowering individuals is lower than that from autumn‐flowering individuals, occasional flowering events may occur in spring (personal observation by the National Park ranger in the Ogasawara Islands). The seeds used in this study were obtained from fruits produced by autumn‐flowering individuals, suggesting that autumn‐flowering seedlings, such as those investigated, are unlikely to be genetically hybridized with neighboring relatives because of the temporal isolation mechanism of the flowering time. The flowering period of *P. parvifolium* is highly differentiated among parapatric *Pittosporum* species, suggesting that it is an important factor in maintaining species integrity. Sugai et al. (2019) investigated the differentiation of flowering periods among three endemic species of the genus *Callicarpa* on Chichijima Island, which was similar to the isolation mechanisms of *Pittosporum*. Temporalisolation, resulting from differences in flowering periods, is the primary mechanism underlying reproductive isolation.

### 4.2 Evaluation of Genetic Parameters Among In Situ and Ex Situ Populations

#### 4.2.1 Wild Individuals of *P. parvifolium*

The genetic diversity of *P. parvifolium* observed in this study was higher than that of other oceanic island endemic species. The wild *P. parvifolium* population was the smallest population size in this study (*N* = 5), with *H*_O_ = 0.60 and *H*_E_ = 0.56. The heightened genetic diversity observed in *P. parvifolium* could be attributed to a relatively recent population decline. The native habitat of *P. parvifolium*, characterized by gentle eastward slopes or scrubs near hilltops or mountain peaks (approximately 300 m above sea level), was subjected to human disturbance following its settlement in the 1870s, approximately 150 years ago. Therefore, *P. parvifolium* has been classified as a critically endangered (CR) species. However, as shown in Table 1, the estimated heterozygosity values, represented by *H*_O_ and *H*_E_, were sufficiently high to support the production of offspring with significant genetic diversity. However, the number of different alleles (*N*_A_ = 3.92) was lower than that of the other two Chichijima *Pittosporum* species. The high level of individual heterozygosity in the HWE and linkage equilibrium of the wild population could be ascribed to the abrupt decrease in the number of wild populations.

In general, the effective population size, Ne, is assumed to be smaller than the actual population size, *N* (Frankham, Ballou, and Briscoe 2006). The larger effective population size observed in this study suggests that events leading to a reduction in genetic diversity, such as bottlenecks, may not have occurred, contrary to expectations, given the small number of existing individuals. This high genetic diversity of wild *P. parvifolium* at the individual level (Figure 2a) and the abrupt reduction in effective population size (Ne) also suggested that previous population sizes were estimated to be high (Figure 2b). The fact that the ancient population size was much larger than that following the HWE corroborates this. In *P. parvifolium*, genetic diversity was maintained despite the small sample size, whereas in studies of the dioecious genus *Robinsonia*, which is endemic to the Juan Fernández Islands, *R. thurifera* showed *H*_O_ = 0.20 and *H*_E_ = 0.10, with a sample size of *N* = 1 (Takayama et al. 2015). This difference in results between these two rare and endemic oceanic island species might be due to the fact that *P. parvifolium* grows in a drier environment, has higher wood density, and a longer generation time, which may contribute to the maintenance of high genetic diversity.

#### 4.2.2 Genetic Characteristics of In Situ Seedlings in 2022 and 2023

The genetic diversity observed in the seedlings of *P. parvifolium* from the 2022 and 2023 seasons, originating from wild individuals A and B, showed high levels of genetic diversity, with observed heterozygosity (*H*_O_) ranging from 0.46 to 0.66 (Table 1). The seeds collected in 2022 from the selfing of wild individual B exhibited lower genetic diversity than those collected in 2023, which was the result of outcrossing between wild individuals A and B. Additionally, individual heterozygosity in the 2023seedlings was significantly higher than that of the 2022 seedlings (Table 1 and Figure 2a), reaching levels comparable to those of the wild population.

To restore species with reduced population sizes, it is crucial to include greater genetic variation. Individuals derived from outcrossing are generally considered to have higher success rates than those obtained from selfing (Frankham, Ballou, and Briscoe 2006). Therefore, as a future conservation strategy, it is desirable to actively utilize seedlings obtained through outcrossing, such as those from the 2023seedlings.

#### 4.2.3 Ex Situ Conservation

The ex situ conservation stocks collected around the time of World Heritage registration in 2011, derived from a limited number of individuals (*N* = 7), exhibited higher‐than‐expected genetic diversity. This notable genetic diversity, exemplified by unique alleles found in the ex situ stocks, may be attributed to the preservation of genetic material from individuals that are now extinct and no longer found in their wild habitats, or it may have resulted from the presence of alleles that have been lost in the current native population (Table S2). Specifically, reintroduction of the seeds back into the in situ population, created by crossing individuals with unique alleles (e.g., k1 and k11) with those that do not have these alleles, could serve as a potential approach.

### 4.3 Conservation Strategies Based on the Present Study

*Pittosporum parvifolium*, a critically endangered species, is one of the most pressing subjects of conservation efforts on the Ogasawara Islands. This species is represented by an extremely limited population of only four individuals, all of which are confined to sclerophyllous shrublands situated in the harshly desiccated conditions of the core area of the World Heritage Site. The precarious existence of *P. parvifolium* underscores its critical conservation status and urgent need for targeted conservation measures to ensure its survival. This study aimed to formulate scientific guidelines for increasing the population size of *Pittosporum parvifolium* while ensuring the maintenance of high genetic diversity based on detailed genetic knowledge and analyses. Notably, only four wild‐type individuals exhibited high levels of heterozygosity and genetic diversity. Additionally, the ex situ population exhibited high levels of heterozygosity and contained unique alleles. Furthermore, our analyses revealed that the past effective population size was larger than the current in situ population size, indicating a rapid decline in individual numbers over the past 150 years. Consequently, it is our responsibility to implement conservation measures aimed at recovering the population size.

In summary, a complementary conservation strategy that combines in situ and ex situ methods is recommended. This strategy includes the introduction of ex situ pollen and/or cuttings from individuals possessing unique alleles into the in situ population to boost the A_R_ and genetic diversity. Furthermore, it is essential to establish a system to track the genetic profiles of wild seedlings and design spatial arrangements for reintroduction that promote genetic diversity through outcrossing.

From an in situ conservation perspective, a notable issue is the production of seedlings via selfing (2022 seedlings). This issue stems from the fact that hermaphroditic individuals (wild B, Figure S2) are self‐compatible, enabling their seeds to propagate and sustain themselves for a minimum of 2 years. The individual heterozygosity (IH, Figure 2a) of the 2022 salfed seedlings was significantly lower than that of the outcrossed 2023seedlings, reflecting a reduction in genetic diversity and increased offspring homogeneity in the wild habitat.

To prevent future genetic issues, the following conservation measures should be implemented: Actively facilitating artificial outcrossing between individuals from geographically distant populations (e.g., wild B × wild D and wild B × wild C, Figure 1c), as this can overcome the limitations of local mating opportunities. Additionally, reinforcement of the in situ population with pollen and seeds from the ex situ population carrying unique alleles is recommended. These measures are intended to enhance both the genetic diversity and population size of in situ populations.

This study provided the first valuable genetic information of a critically endangered species with only four extant individuals that have left the problem alone for nearly 30 years. As a woody species, *P. parvifolium* is expected to need considerable time to be mature, making it challenging to restore and stabilize its population rapidly. The government needs to maintain consistent in situ and ex situ conservation efforts over an extended period. Ensuring the long‐term conservation of a species with a critically low population of only four individuals necessitates the adoption of a patient, evidence‐based approach informed by the findings of this study.

## Author Contributions

**Haruna Kawakita:** conceptualization (lead), data curation (lead), formal analysis (lead), investigation (lead), methodology (lead), project administration (lead), visualization (lead), writing – original draft (lead), writing – review and editing (lead). **Shota Sakaguchi:** data curation (supporting), formal analysis (supporting), methodology (equal), supervision (supporting), writing – original draft (supporting), writing – review and editing (supporting). **Saeko Katoh:** resources (supporting). **Hidetoshi Kato:** resources (supporting). **Takefumi Tanaka:** resources (supporting). **Yoshiteru Komaki:** resources (supporting). **Takahito Ideno:** investigation (supporting), resources (supporting). **Hiroaki Setoguchi:** conceptualization (supporting), funding acquisition (lead), investigation (supporting), methodology (supporting), supervision (lead), writing – original draft (supporting), writing – review and editing (supporting).

## Conflicts of Interest

The authors declare no conflicts of interest.

## Supporting information


**Figure S1.** Additional details regarding the habitat and characteristics of *Pittosporum parvifolium*: (a) The habitat scenery of *P. parvifolium*, characterized by a dry scrub forest environment. (b) A wild individual of *P. parvifolium* enclosed and protected by a fence cage. (c) Seeds and dehiscent capsules of *P. parvifolium*. (d) Appearance of ex situ conservation at a botanical garden.

**Figure S2.** Two types of flowers of *Pittosporum parvifolium*. (a) Male flower with degenerated anthers. (b) Hermaphrodite flower. Front petals are removed to show anthers and pistils.

**Table S1.** Details of 12 microsatellite markers developed by Katoh et al. (2013) and used in this study. Ta: the annealing temperature of the primer pair, *A*: number of alleles per locus, *H*_O_: observed heterozygosity, *H*_E_: expected heterozygosity * Significant deviation from HWE expectations (*p* < 0.05).

**Table S2.** Genotype data of whole samples (*N* = 144) with 12 SSR makers. In terms of pop “ex situ,” a locus with *, **, *** means three unique alleles which only ex situ individuals have. The column “pop” abbreviation PP, PB, PC, and PH mean *P. parvifolium*, *P. boninense*, *P. chichijimense*, and *P. beecheyi,* respectively.

## Data Availability

All data are available in the main text or in the Appendix S1.
