# Supplementary figures and images for: Potential of Ex Situ Conservation Strains Revealed by Genetic Analysis of Oceanic Islands' Endangered Species Pittosporum parvifolium
Source: Ecol Evol. 2024 Oct 30;14(11):e70506. doi: 10.1002/ece3.70506 (PMC11522610; doi:10.1002/ece3.70506)

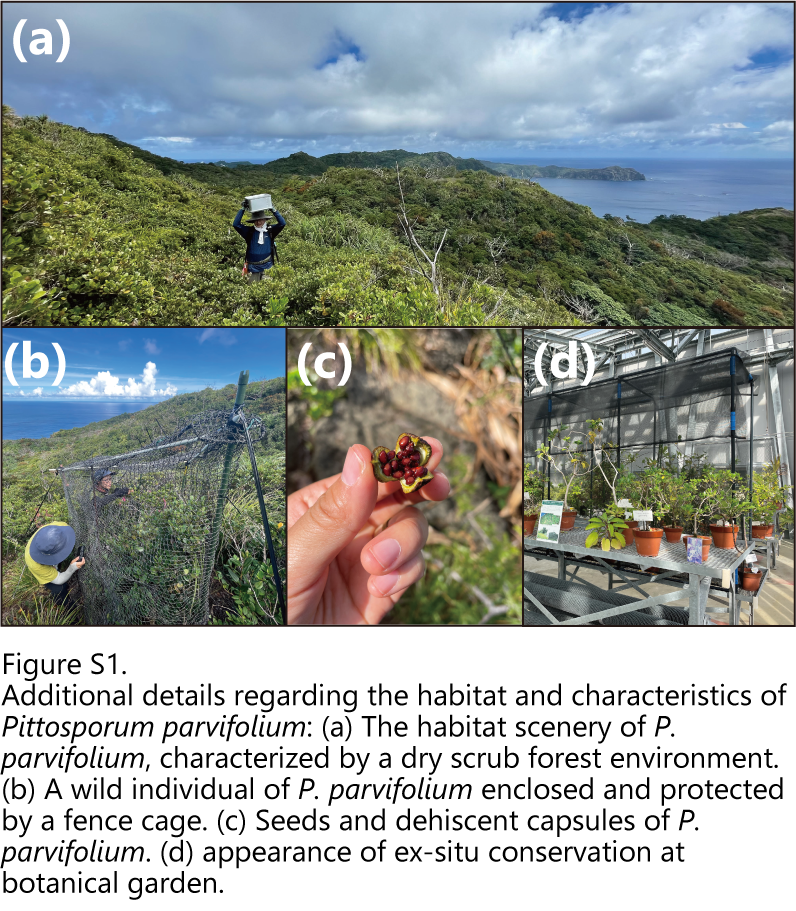

Supplement: Supplementary file 1 — Figure S1. Additional details regarding the habitat and characteristics of Pittosporum parvifolium: (a) The habitat scenery of P. parvifolium, characterized by a dry scrub forest environment. (b) A wild individual of P. parvifolium enclosed and protected by a fence cage. (c) Seeds and dehiscent capsules of P. parvifolium. (d) Appearance of ex situ conservation at a botanical garden. [file ECE3-14-e70506-s001.tif]

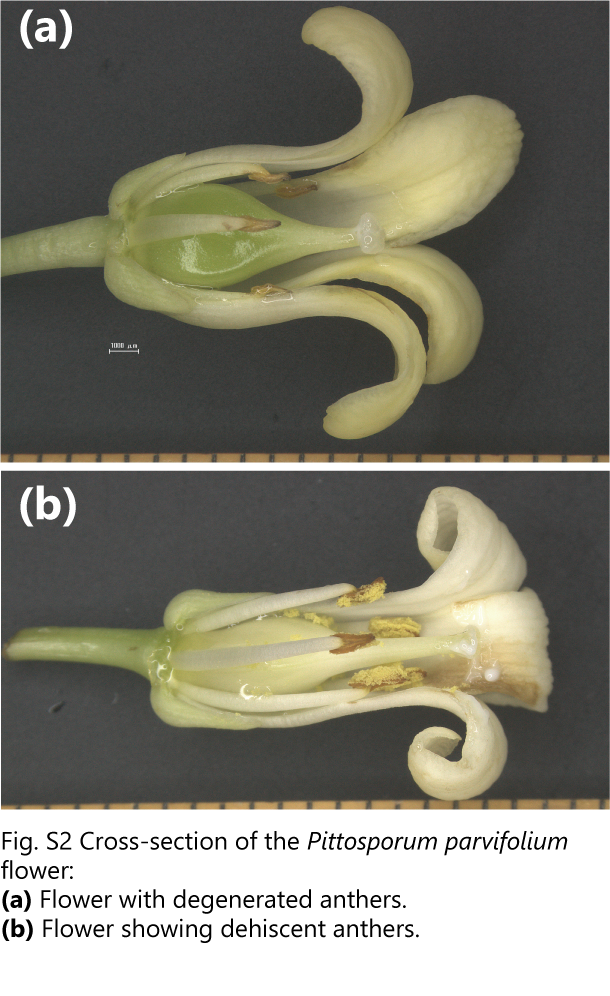

Supplement: Supplementary file 2 — Figure S2. Two types of flowers of Pittosporum parvifolium. (a) Male flower with degenerated anthers. (b) Hermaphrodite flower. Front petals are removed to show anthers and pistils. [file ECE3-14-e70506-s004.tif]
