# Supplementary material for: Potential of Ex Situ Conservation Strains Revealed by Genetic Analysis of Oceanic Islands' Endangered Species Pittosporum parvifolium
Source: Ecol Evol. 2024 Oct 30;14(11):e70506. doi: 10.1002/ece3.70506 (PMC11522610; doi:10.1002/ece3.70506)
Supplement: Supplementary file 3 — Table S1. Details of 12 microsatellite markers developed by Katoh et al. (2013) and used in this study. Ta: the annealing temperature of the primer pair, A: number of alleles per locus, H O: observed heterozygosity, H E: expected heterozygosity * Significant deviation from HWE expectations (p < 0.05). [file ECE3-14-e70506-s003.docx]

Kawakita et al., 2024 Supplementary materials

Table S1. Details of 12 microsatellite markers selected from Katoh et al. (2013) and used in this study

| Locus | Repeat motif | Primer sequence (5'-3') | Allele size range (bp) | *T*a (℃) | Accession No. | *A* | *H*_O_ | *H*_E_ |
| --- | --- | --- | --- | --- | --- | --- | --- | --- |
| PP14010 | (AC)6(TC)14 | ACACACACACACTCTCTCTCTC | 75–103 | 58 | AB699136 | 4 | 0.86 | 0.69 |
|  |  | CATTTTAGTCTAGGCTTCAAGAACA |  |  |  |  |  |  |
| PB14020 | (AC)6(TC)5 | ACACACACACACTCTCTCTCTC | 173–271 | 60 | AB698927 | 15 | 0.45* | 0.81 |
|  |  | CGCAAACACAATACCTGGAA |  |  |  |  |  |  |
| PB14027 | (AC)6(TC)15 | ACACACACACACTCTCTCTCTC | 159–197 | 60 | AB698928 | 16 | 0.88 | 0.92 |
|  |  | CTTTGCAGATTCGCAACAAC |  |  |  |  |  |  |
| PB14031 | (AC)6(TC)6 | ACACACACACACTCTCTCTCTC | 231–319 | 60 | AB698929 | 23 | 0.52* | 0.94 |
|  |  | CTTGCCCTCTGATTCCAAAA |  |  |  |  |  |  |
| PP12016 | (TC)6(AC)11 | TCTCTCTCTCTCACACACACAC | 133–137 | 60 | AB699134 | 2 | 0.43 | 0.34 |
|  |  | GGAGCCAAGGTTCCTAAAAGA |  |  |  |  |  |  |
| PB11009 | (AC)6(AG)5 | ACACACACACACAGAGAGAGAG | 80–90 | 59 | AB698886 | 5 | 0.58 | 0.59 |
|  |  | GTTTTGGCAGAATGTGATGC |  |  |  |  |  |  |
| PB21002 | (AC)6(AG)7 | ACACACACACACAGAGAGAGAG | 69–97 | 58 | AB698934 | 7 | 0.21* | 0.51 |
|  |  | ATTCTTTGCACTGCTAAACA |  |  |  |  |  |  |
| PB22012 | (TC)6(AC)14 | TCTCTCTCTCTCACACACACAC | 162–168 | 57 | AB698940 | 4 | 0.46 | 0.61 |
|  |  | GGGAAATTGCTAGTTATAAGA |  |  |  |  |  |  |
| PB22015 | (TC)6(AC)16 | TCTCTCTCTCTCACACACACAC | 61–79 | 55 | AB698941 | 10 | 0.88 | 0.86 |
|  |  | AAGAACACTGAAAGATATCCT |  |  |  |  |  |  |
| PH11121 | (AC)6(AG)5 | ACACACACACACAGAGAGAGAG | 166–182 | 60 | AB698957 | 4 | 0.38 | 0.55 |
|  |  | TCCCCCATATTCATTTTGGA |  |  |  |  |  |  |
| PH11145 | (AC)6(AG)9 | ACACACACACACAGAGAGAGAG | 201–227 | 60 | AB698958 | 5 | 0.88 | 0.79 |
|  |  | GCAGAGCCTTATTTGGATCG |  |  |  |  |  |  |

*T*a, annealing temperature of the primer pair; *A*, number of alleles per locus; *H*_O_, observed heterozygosity; *H*_E_, expected heterozygosity * Significant deviation from HWE expectations (*p* < 0.05)
