# Supplementary material for: Potential of Ex Situ Conservation Strains Revealed by Genetic Analysis of Oceanic Islands' Endangered Species Pittosporum parvifolium
Source: Ecol Evol. 2024 Oct 30;14(11):e70506. doi: 10.1002/ece3.70506 (PMC11522610; doi:10.1002/ece3.70506)
Supplement: Supplementary file 4 — Table S2. Genotype data of whole samples (N = 144) with 12 SSR makers. In terms of pop “ex situ,” a locus with *, **, *** means three unique alleles which only ex situ individuals have. The column “pop” abbreviation PP, PB, PC, and PH mean P. parvifolium, P. boninense, P. chichijimense, and P. beecheyi, respectively. [file ECE3-14-e70506-s002.docx]

Kawakita et al., 2024 Supplementary Materials

Table S2. Genotyping data of whole samples (N=144) with 12 SSR makers. In terms of pop "ex-situ", locus with *, **, *** mean three unique alleles which only ex-situ individuals have. The column "pop" abbreviation PP, PB, PC, and PH mean *P. parvifolium*, *P. boninense*, *P. chichijimense*, and *P. beecheyi* respectively.

| sample ID | pop | No1  PP14010 | | No4  PB14020 | | No5  PB14027 | | No6  PB14031 | | No11  PP12016 | | No18  PB11009 | | No19  PB21002 | | No21  PB22012 | | No22  PB22015 | | No23  PH11121 | | No24  PH11145 | | No25  PH14003 | |
| --- | --- | --- | --- | --- | --- | --- | --- | --- | --- | --- | --- | --- | --- | --- | --- | --- | --- | --- | --- | --- | --- | --- | --- | --- | --- |
| Wild E | PP | 68 | 90 | 219 | 227 | 154 | 162 | 268 | 278 | 134 | 134 | 79 | 85 | 85 | 89 | 168 | 168 | 66 | 66 | 173 | 173 | 206 | 208 | 178 | 178 |
| Wild A | PP | 68 | 90 | 222 | 237 | 154 | 160 | 271 | 286 | 130 | 134 | 79 | 85 | 85 | 89 | 166 | 170 | 64 | 64 | 173 | 173 | 208 | 208 | 178 | 178 |
| Wild B | PP | 90 | 94 | 219 | 249 | 154 | 156 | 268 | 298 | 130 | 130 | 79 | 85 | 87 | 89 | 162 | 170 | 64 | 64 | 173 | 177 | 206 | 208 | 168 | 178 |
| Wild C | PP | 68 | 68 | 207 | 237 | 154 | 158 | 256 | 286 | 130 | 134 | 79 | 79 | 89 | 89 | 166 | 166 | 66 | 66 | 173 | 177 | 208 | 208 | 178 | 184 |
| Wild D | PP | 90 | 98 | 252 | 257 | 139 | 156 | 302 | 306 | 130 | 130 | 79 | 79 | 89 | 97 | 166 | 166 | 66 | 66 | 173 | 173 | 208 | 208 | 178 | 178 |
| k1 | ex-situ | 90 | 94 | 219 | 252 | 156 | 156 | 268 | 302 | 130 | 130 | 79 | 85 | 87 | 97 | 162 | 166 | 66 | 66 | 177 | 177 | 208 | 208 | 168 | 178 |
| k7 | ex-situ | 68 | 90 | 227 | 249 | 158 | 162 | 278 | 300* | 130 | 134 | 85 | 85 | 85 | 89 | 168 | 170 | 66 | 66 | 173 | 177 | 206 | 206 | 178 | 178 |
| k8 | ex-situ | 68 | 68 | 227 | 249 | 154 | 158 | 278 | 300* | 130 | 134 | 79 | 85 | 89 | 89 | 166 | 168 | 66 | 66 | 173 | 177 | 206 | 208 | 178 | 178 |
| k10 | ex-situ | 68 | 94 | 237 | 249 | 154 | 156 | 286 | 300* | 130 | 130 | 79 | 85 | 85 | 89 | 166 | 166 | 64 | 64 | 169 | 173 | 206 | 208 | 178 | 178 |
| k11 | ex-situ | 68 | 68 | 210*** | 227 | 158 | 162 | 260** | 278 | 130 | 134 | 85 | 85 | 89 | 89 | 168 | 170 | 66 | 66 | 173 | 177 | 206 | 206 | 178 | 178 |
| k12 | ex-situ | 68 | 94 | 219 | 249 | 154 | 156 | 268 | 300* | 130 | 134 | 85 | 85 | 85 | 89 | 168 | 170 | 66 | 66 | 173 | 177 | 206 | 206 | 178 | 178 |
| k13 | ex-situ | 94 | 94 | 219 | 249 | 154 | 156 | 268 | 300* | 130 | 130 | 85 | 85 | 87 | 89 | 162 | 170 | 64 | 64 | 173 | 177 | 206 | 208 | 168 | 178 |
| k14 | ex-situ | 68 | 94 | 222 | 249 | 154 | 156 | 271 | 300* | 130 | 130 | 79 | 85 | 89 | 89 | 166 | 170 | 64 | 64 | 173 | 177 | 206 | 208 | 178 | 178 |
| k15 | ex-situ | 68 | 90 | 210*** | 227 | 158 | 162 | 260** | 278 | 130 | 134 | 79 | 85 | 85 | 89 | 166 | 168 | 66 | 66 | 173 | 173 | 206 | 208 | 178 | 178 |
| k16 | ex-situ | 68 | 94 | 227 | 249 | 154 | 156 | 278 | 300* | 130 | 134 | 85 | 85 | 89 | 89 | 166 | 168 | 66 | 66 | 173 | 173 | 206 | 208 | 178 | 178 |
| k17 | ex-situ | 68 | 90 | 210*** | 219 | 158 | 162 | 260** | 268 | 130 | 134 | 85 | 85 | 89 | 89 | 166 | 168 | 66 | 66 | 173 | 173 | 206 | 206 | 178 | 178 |
| k18 | ex-situ | 90 | 98 | 219 | 252 | 139 | 156 | 268 | 302 | 130 | 130 | 79 | 85 | 87 | 97 | 166 | 170 | 64 | 64 | 173 | 177 | 206 | 208 | 178 | 178 |
| k19 | ex-situ | 90 | 98 | 249 | 252 | 139 | 156 | 298 | 302 | 130 | 130 | 79 | 85 | 87 | 89 | 166 | 170 | 64 | 64 | 173 | 173 | 206 | 208 | 178 | 178 |
| k20 | ex-situ | 90 | 90 | 219 | 252 | 156 | 156 | 268 | 302 | 130 | 130 | 79 | 79 | 87 | 89 | 166 | 170 | 64 | 64 | 173 | 177 | 206 | 208 | 178 | 178 |
| k21 | ex-situ | 90 | 98 | 219 | 252 | 139 | 156 | 268 | 302 | 130 | 130 | 79 | 79 | 87 | 89 | 162 | 166 | 66 | 66 | 177 | 177 | 208 | 208 | 178 | 178 |
| k22 | ex-situ | 90 | 94 | 219 | 257 | 154 | 156 | 268 | 306 | 130 | 130 | 79 | 79 | 89 | 97 | 166 | 170 | 64 | 64 | 177 | 177 | 208 | 208 | 178 | 178 |
| k23 | ex-situ | 90 | 98 | 249 | 257 | 139 | 156 | 298 | 306 | 130 | 130 | 79 | 85 | 87 | 97 | 162 | 166 | 64 | 64 | 177 | 177 | 208 | 208 | 168 | 178 |
| k24 | ex-situ | 90 | 90 | 249 | 257 | 156 | 156 | 298 | 306 | 130 | 130 | 79 | 79 | 87 | 89 | 166 | 170 | 66 | 66 | 177 | 177 | 208 | 208 | 168 | 178 |
| k25 | ex-situ | 94 | 98 | 249 | 257 | 139 | 154 | 298 | 306 | 130 | 130 | 79 | 79 | 89 | 97 | 166 | 170 | 64 | 64 | 173 | 177 | 208 | 208 | 178 | 178 |
| k27 | ex-situ | 90 | 94 | 227 | 249 | 156 | 162 | 278 | 300* | 130 | 134 | 85 | 85 | 89 | 89 | 166 | 168 | 66 | 66 | 173 | 177 | 206 | 206 | 178 | 178 |
| k28 | ex-situ | 68 | 94 | 210*** | 227 | 154 | 156 | 260** | 278 | 130 | 134 | 85 | 85 | 89 | 89 | 168 | 170 | 66 | 66 | 173 | 173 | 206 | 206 | 178 | 178 |
| k29 | ex-situ | 68 | 94 | 210*** | 219 | 154 | 156 | 260** | 268 | 130 | 134 | 85 | 85 | 89 | 89 | 166 | 168 | 66 | 66 | 173 | 173 | 206 | 206 | 178 | 178 |
| k30 | ex-situ | 90 | 94 | 210*** | 210*** | 156 | 162 | 260** | 260** | 130 | 134 | 85 | 85 | 89 | 89 | 168 | 168 | 66 | 66 | 173 | 173 | 206 | 208 | 178 | 178 |
| k31 | ex-situ | 68 | 68 | 227 | 249 | 154 | 158 | 278 | 300* | 130 | 134 | 85 | 85 | 89 | 89 | 166 | 168 | 66 | 66 | 173 | 173 | 206 | 208 | 178 | 178 |
| k32 | ex-situ | 68 | 90 | 227 | 249 | 156 | 162 | 278 | 300* | 130 | 134 | 85 | 85 | 89 | 89 | 168 | 170 | 66 | 66 | 173 | 173 | 206 | 206 | 178 | 178 |
| k33 | ex-situ | 94 | 94 | 249 | 275 | 156 | 156 | 300* | 300* | 134 | 134 | 85 | 85 | 85 | 89 | 166 | 168 | 66 | 66 | 173 | 173 | 208 | 208 | 178 | 178 |
| k34 | ex-situ | 68 | 90 | 249 | 252 | 156 | 158 | 300* | 302 | 130 | 130 | 79 | 85 | 89 | 97 | 166 | 170 | 66 | 66 | 173 | 173 | 206 | 208 | 178 | 178 |
| k35 | ex-situ | 68 | 90 | 249 | 257 | 156 | 158 | 300* | 306 | 130 | 130 | 79 | 85 | 89 | 89 | 166 | 170 | 66 | 66 | 177 | 177 | 206 | 208 | 178 | 178 |
| k36 | ex-situ | 94 | 98 | 249 | 252 | 139 | 156 | 300* | 302 | 130 | 130 | 79 | 85 | 89 | 89 | 166 | 170 | 66 | 66 | 173 | 173 | 206 | 208 | 178 | 178 |
| k37 | ex-situ | 94 | 98 | 210*** | 252 | 139 | 158 | 260** | 302 | 130 | 130 | 79 | 85 | 89 | 97 | 166 | 166 | 66 | 66 | 173 | 173 | 206 | 208 | 178 | 178 |
| k38 | ex-situ | 90 | 94 | 210*** | 252 | 156 | 156 | 260** | 302 | 130 | 130 | 79 | 85 | 89 | 89 | 166 | 170 | 66 | 66 | 173 | 177 | 206 | 208 | 178 | 178 |
| seedling1 | 2022seedlings | 90 | 94 | 249 | 249 | 154 | 156 | 298 | 298 | 130 | 130 | 85 | 85 | 87 | 89 | 162 | 170 | 64 | 64 | 177 | 177 | 206 | 206 | 168 | 178 |
| seedling2 | 2022seedlings | 90 | 90 | 219 | 249 | 156 | 156 | 268 | 298 | 130 | 130 | 79 | 79 | 87 | 89 | 162 | 170 | 64 | 64 | 173 | 173 | 206 | 206 | 178 | 178 |
| seedling3 | 2022seedlings | 94 | 94 | 249 | 249 | 154 | 156 | 298 | 298 | 130 | 130 | 79 | 85 | 87 | 89 | 162 | 170 | 64 | 64 | 173 | 177 | 208 | 208 | 168 | 178 |
| seedling4 | 2022seedlings | 90 | 94 | 219 | 249 | 154 | 156 | 268 | 298 | 130 | 130 | 79 | 79 | 87 | 89 | 162 | 170 | 64 | 64 | 177 | 177 | 206 | 206 | 168 | 178 |
| seedling5 | 2022seedlings | 90 | 94 | 219 | 249 | 154 | 156 | 268 | 298 | 130 | 130 | 79 | 79 | 87 | 89 | 162 | 170 | 64 | 64 | 173 | 177 | 206 | 206 | 168 | 178 |
| seedling6 | 2022seedlings | 90 | 94 | 219 | 249 | 154 | 156 | 268 | 298 | 130 | 130 | 79 | 79 | 87 | 87 | 162 | 170 | 64 | 64 | 173 | 173 | 208 | 208 | 168 | 178 |
| seedling7 | 2022seedlings | 90 | 90 | 219 | 249 | 156 | 156 | 268 | 298 | 130 | 130 | 79 | 79 | 89 | 89 | 162 | 170 | 64 | 64 | 173 | 177 | 206 | 208 | 168 | 178 |
| seedling8 | 2022seedlings | 90 | 94 | 219 | 249 | 154 | 156 | 268 | 298 | 130 | 130 | 79 | 79 | 89 | 89 | 162 | 162 | 64 | 64 | 173 | 177 | 208 | 208 | 168 | 178 |
| seedling9 | 2022seedlings | 90 | 94 | 219 | 249 | 154 | 156 | 268 | 298 | 130 | 130 | 85 | 85 | 87 | 89 | 162 | 170 | 64 | 64 | 177 | 177 | 206 | 208 | 168 | 168 |
| seedling10 | 2022seedlings | 90 | 90 | 219 | 249 | 156 | 156 | 268 | 268 | 130 | 130 | 85 | 85 | 87 | 89 | 162 | 170 | 64 | 64 | 173 | 177 | 206 | 208 | 168 | 178 |
| seedling11 | 2022seedlings | 90 | 94 | 249 | 249 | 154 | 156 | 298 | 298 | 130 | 130 | 79 | 79 | 87 | 89 | 162 | 170 | 64 | 64 | 173 | 173 | 206 | 206 | 168 | 178 |
| seedling12 | 2022seedlings | 90 | 94 | 219 | 219 | 154 | 156 | 268 | 268 | 130 | 130 | 79 | 79 | 87 | 87 | 162 | 170 | 64 | 64 | 177 | 177 | 206 | 208 | 168 | 178 |
| seedling13 | 2022seedlings | 90 | 94 | 219 | 219 | 154 | 156 | 268 | 268 | 130 | 130 | 79 | 85 | 87 | 89 | 162 | 170 | 64 | 64 | 173 | 177 | 206 | 208 | 178 | 178 |
| seedling14 | 2022seedlings | 90 | 94 | 219 | 249 | 154 | 154 | 268 | 298 | 130 | 130 | 85 | 85 | 87 | 89 | 162 | 170 | 64 | 64 | 173 | 177 | 206 | 208 | 168 | 178 |
| seedling15 | 2022seedlings | 90 | 90 | 219 | 219 | 156 | 156 | 268 | 268 | 130 | 130 | 79 | 85 | 87 | 89 | 162 | 162 | 64 | 64 | 177 | 177 | 206 | 206 | 178 | 178 |
| seedling16 | 2022seedlings | 90 | 94 | 219 | 249 | 154 | 156 | 268 | 298 | 130 | 130 | 79 | 79 | 89 | 89 | 162 | 170 | 64 | 64 | 173 | 177 | 206 | 208 | 168 | 178 |
| seedling17 | 2022seedlings | 90 | 90 | 219 | 249 | 156 | 156 | 268 | 298 | 130 | 130 | 79 | 79 | 89 | 89 | 170 | 170 | 64 | 64 | 173 | 177 | 208 | 208 | 168 | 168 |
| seedling18 | 2022seedlings | 94 | 94 | 219 | 219 | 154 | 154 | 268 | 268 | 130 | 130 | 85 | 85 | 87 | 89 | 162 | 170 | 64 | 64 | 173 | 173 | 206 | 206 | 178 | 178 |
| seedling19 | 2022seedlings | 90 | 94 | 219 | 249 | 154 | 156 | 268 | 298 | 130 | 130 | 79 | 85 | 87 | 87 | 162 | 162 | 64 | 64 | 173 | 177 | 206 | 206 | 168 | 178 |
| seedling20 | 2022seedlings | 94 | 94 | 249 | 249 | 154 | 154 | 298 | 298 | 130 | 130 | 79 | 85 | 87 | 89 | 162 | 162 | 64 | 64 | 173 | 177 | 206 | 208 | 168 | 178 |
| seedling21 | 2022seedlings | 90 | 94 | 249 | 249 | 156 | 156 | 268 | 298 | 130 | 130 | 79 | 85 | 87 | 89 | 162 | 162 | 64 | 64 | 173 | 177 | 208 | 208 | 166 | 166 |
| seedling22 | 2022seedlings | 90 | 90 | 249 | 249 | 156 | 156 | 298 | 298 | 130 | 130 | 79 | 85 | 87 | 89 | 162 | 170 | 64 | 64 | 173 | 177 | 206 | 208 | 168 | 178 |
| seedling23 | 2022seedlings | 90 | 94 | 219 | 249 | 154 | 156 | 298 | 298 | 130 | 130 | 79 | 79 | 87 | 89 | 162 | 170 | 64 | 64 | 177 | 177 | 208 | 208 | 168 | 168 |
| seedling24 | 2022seedlings | 90 | 94 | 219 | 249 | 154 | 156 | 268 | 298 | 130 | 130 | 79 | 85 | 89 | 89 | 162 | 170 | 64 | 64 | 173 | 173 | 206 | 208 | 168 | 178 |
| seedling25 | 2022seedlings | 90 | 94 | 249 | 249 | 154 | 154 | 268 | 298 | 130 | 130 | 79 | 85 | 89 | 89 | 162 | 170 | 64 | 64 | 173 | 177 | 206 | 207 | 178 | 178 |
| seedling26 | 2022seedlings | 94 | 94 | 219 | 249 | 154 | 154 | 298 | 298 | 130 | 130 | 85 | 85 | 87 | 89 | 162 | 162 | 64 | 64 | 173 | 173 | 206 | 206 | 166 | 176 |
| seedling27 | 2022seedlings | 90 | 90 | 219 | 249 | 156 | 156 | 268 | 298 | 130 | 130 | 79 | 85 | 89 | 89 | 162 | 170 | 64 | 64 | 173 | 173 | 206 | 207 | 178 | 178 |
| seedling28 | 2022seedlings | 90 | 90 | 249 | 249 | 156 | 156 | 268 | 298 | 130 | 130 | 79 | 85 | 87 | 89 | 162 | 162 | 64 | 64 | 173 | 173 | 206 | 208 | 168 | 178 |
| seedling29 | 2022seedlings | 90 | 90 | 219 | 249 | 156 | 156 | 298 | 298 | 130 | 130 | 79 | 85 | 87 | 89 | 162 | 170 | 64 | 64 | 177 | 177 | 206 | 206 | 178 | 178 |
| seedling30 | 2022seedlings | 90 | 90 | 219 | 219 | 156 | 156 | 268 | 298 | 130 | 130 | 79 | 85 | 87 | 89 | 170 | 170 | 64 | 64 | 173 | 177 | 208 | 208 | 168 | 178 |
| seedling31 | 2022seedlings | 94 | 94 | 219 | 249 | 154 | 156 | 268 | 268 | 130 | 130 | 79 | 79 | 87 | 89 | 162 | 162 | 64 | 64 | 177 | 177 | 206 | 208 | 168 | 168 |
| seedling32 | 2022seedlings | 90 | 94 | 249 | 249 | 154 | 154 | 268 | 298 | 130 | 130 | 79 | 85 | 87 | 87 | 170 | 170 | 64 | 64 | 173 | 173 | 208 | 208 | 168 | 168 |
| seedling33 | 2022seedlings | 90 | 90 | 219 | 249 | 156 | 156 | 298 | 298 | 130 | 130 | 79 | 85 | 87 | 87 | 170 | 170 | 64 | 64 | 173 | 177 | 206 | 208 | 168 | 168 |
| seedling34 | 2022seedlings | 90 | 94 | 219 | 249 | 154 | 156 | 268 | 298 | 130 | 130 | 79 | 79 | 87 | 89 | 162 | 170 | 64 | 64 | 173 | 177 | 206 | 208 | 178 | 178 |
| seedling35 | 2022seedlings | 90 | 94 | 219 | 249 | 154 | 156 | 268 | 298 | 130 | 130 | 79 | 79 | 87 | 87 | 162 | 170 | 64 | 64 | 173 | 173 | 206 | 208 | 166 | 176 |
| seedling36 | 2022seedlings | 90 | 94 | 219 | 249 | 154 | 156 | 268 | 298 | 130 | 130 | 79 | 85 | 87 | 89 | 170 | 170 | 64 | 64 | 173 | 177 | 208 | 208 | 168 | 178 |
| seedling37 | 2022seedlings | 90 | 94 | 219 | 219 | 154 | 156 | 268 | 298 | 130 | 130 | 79 | 85 | 87 | 87 | 162 | 162 | 64 | 64 | 173 | 177 | 206 | 208 | 168 | 168 |
| 2ndPPs_1 | 2023seedlings | 90 | 94 | 219 | 222 | 156 | 160 | 268 | 271 | 130 | 130 | 85 | 85 | 89 | 89 | 162 | 170 | 64 | 64 | 173 | 177 | 208 | 208 | 168 | 178 |
| 2ndPPs_2 | 2023seedlings | 68 | 90 | 219 | 237 | 154 | 154 | 268 | 286 | 130 | 130 | 79 | 85 | 85 | 89 | 166 | 170 | 64 | 64 | 173 | 177 | 208 | 208 | 168 | 178 |
| 2ndPPs_3 | 2023seedlings | 68 | 90 | 219 | 222 | 154 | 156 | 268 | 271 | 130 | 134 | 79 | 85 | 87 | 89 | 166 | 170 | 64 | 66 | 173 | 173 | 208 | 208 | 168 | 178 |
| 2ndPPs_4 | 2023seedlings | 90 | 90 | 219 | 222 | 156 | 160 | 268 | 271 | 130 | 130 | 79 | 79 | 85 | 87 | 162 | 170 | 66 | 66 | 173 | 173 | 208 | 208 | 168 | 178 |
| 2ndPPs_5 | 2023seedlings | 68 | 94 | 237 | 249 | 154 | 154 | 286 | 298 | 130 | 130 | 79 | 85 | 87 | 89 | 166 | 170 | 66 | 66 | 173 | 173 | 208 | 208 | 168 | 178 |
| 2ndPPs_6 | 2023seedlings | 90 | 94 | 219 | 237 | 154 | 160 | 268 | 286 | 130 | 134 | 79 | 85 | 85 | 89 | 170 | 170 | 64 | 64 | 173 | 177 | 208 | 208 | 178 | 178 |
| 2ndPPs_7 | 2023seedlings | 90 | 90 | 219 | 237 | 154 | 156 | 268 | 286 | 130 | 134 | 79 | 85 | 87 | 89 | 162 | 166 | 66 | 66 | 173 | 173 | 206 | 208 | 168 | 178 |
| 2ndPPs_8 | 2023seedlings | 68 | 94 | 222 | 249 | 154 | 154 | 271 | 298 | 130 | 134 | 79 | 79 | 85 | 87 | 162 | 170 | 66 | 66 | 173 | 173 | 206 | 208 | 178 | 178 |
| 2ndPPs_9 | 2023seedlings | 90 | 94 | 219 | 237 | 154 | 154 | 268 | 286 | 130 | 134 | 85 | 85 | 85 | 89 | 166 | 170 | 64 | 66 | 173 | 173 | 208 | 208 | 168 | 178 |
| 2ndPPs_10 | 2023seedlings | 68 | 90 | 237 | 249 | 154 | 156 | 286 | 298 | 130 | 134 | 79 | 79 | 89 | 89 | 162 | 166 | 66 | 66 | 177 | 177 | 208 | 208 | 168 | 178 |
| 2ndPPs_11 | 2023seedlings | 68 | 94 | 219 | 237 | 154 | 160 | 268 | 286 | 130 | 134 | 79 | 79 | 85 | 89 | 162 | 170 | 64 | 66 | 173 | 173 | 208 | 208 | 168 | 178 |
| 2ndPPs_12 | 2023seedlings | 68 | 94 | 237 | 249 | 154 | 154 | 286 | 298 | 130 | 134 | 79 | 85 | 85 | 87 | 170 | 170 | 64 | 66 | 173 | 173 | 206 | 208 | 178 | 178 |
| 2ndPPs_13 | 2023seedlings | 90 | 94 | 219 | 237 | 154 | 160 | 268 | 286 | 130 | 134 | 79 | 79 | 87 | 89 | 170 | 170 | 64 | 66 | 173 | 173 | 206 | 208 | 178 | 178 |
| 2ndPPs_14 | 2023seedlings | 90 | 94 | 219 | 222 | 154 | 160 | 268 | 271 | 130 | 130 | 79 | 79 | 85 | 89 | 170 | 170 | 64 | 66 | 177 | 177 | 206 | 208 | 178 | 178 |
| 2ndPPs_15 | 2023seedlings | 90 | 90 | 222 | 249 | 154 | 156 | 271 | 298 | 130 | 134 | 79 | 85 | 87 | 89 | 162 | 170 | 66 | 66 | 173 | 173 | 206 | 208 | 176 | 176 |
| 2ndPPs_16 | 2023seedlings | 68 | 94 | 219 | 222 | 154 | 154 | 268 | 271 | 130 | 134 | 79 | 85 | 85 | 89 | 166 | 170 | 64 | 64 | 173 | 173 | 208 | 208 | 168 | 178 |
| 2ndPPs_17 | 2023seedlings | 68 | 90 | 222 | 249 | 154 | 156 | 271 | 298 | 130 | 134 | 79 | 79 | 85 | 87 | 162 | 170 | 66 | 66 | 173 | 173 | 208 | 208 | 168 | 178 |
| 2ndPPs_18 | 2023seedlings | 68 | 94 | 219 | 222 | 154 | 154 | 268 | 271 | 130 | 134 | 79 | 85 | 89 | 89 | 162 | 170 | 64 | 66 | 173 | 173 | 208 | 208 | 168 | 178 |
| 2ndPPs_19 | 2023seedlings | 68 | 94 | 219 | 237 | 154 | 154 | 268 | 286 | 130 | 134 | 79 | 85 | 89 | 89 | 162 | 166 | 64 | 64 | 177 | 177 | 206 | 208 | 178 | 178 |
| 2ndPPs_20 | 2023seedlings | 68 | 94 | 219 | 237 | 154 | 160 | 268 | 286 | 130 | 130 | 79 | 85 | 85 | 89 | 170 | 170 | 64 | 64 | 173 | 173 | 208 | 208 | 168 | 178 |
| 2ndPPs_21 | 2023seedlings | 90 | 94 | 237 | 249 | 154 | 160 | 286 | 298 | 130 | 130 | 79 | 85 | 87 | 89 | 162 | 166 | 64 | 64 | 173 | 173 | 206 | 208 | 178 | 178 |
| 2ndPPs_22 | 2023seedlings | 68 | 94 | 237 | 249 | 154 | 154 | 286 | 298 | 130 | 134 | 79 | 85 | 89 | 89 | 170 | 170 | 64 | 64 | 177 | 177 | 208 | 208 | 168 | 178 |
| 2ndPPs_23 | 2023seedlings | 90 | 94 | 219 | 237 | 154 | 160 | 268 | 286 | 130 | 130 | 79 | 85 | 85 | 87 | 162 | 166 | 64 | 66 | 173 | 173 | 206 | 208 | 178 | 178 |
| 2ndPPs_24 | 2023seedlings | 90 | 90 | 222 | 249 | 156 | 160 | 271 | 298 | 130 | 130 | 79 | 79 | 87 | 89 | 162 | 170 | 64 | 66 | 177 | 177 | 208 | 208 | 168 | 178 |
| 2ndPPs_25 | 2023seedlings | 68 | 90 | 222 | 249 | 154 | 156 | 271 | 298 | 130 | 134 | 79 | 85 | 89 | 89 | 162 | 166 | 66 | 66 | 173 | 173 | 208 | 208 | 176 | 176 |
| 2ndPPs_26 | 2023seedlings | 68 | 90 | 219 | 237 | 154 | 156 | 268 | 286 | 130 | 134 | 79 | 85 | 89 | 89 | 162 | 166 | 64 | 66 | 173 | 177 | 206 | 208 | 168 | 178 |
| 2ndPPs_27 | 2023seedlings | 90 | 94 | 222 | 249 | 154 | 160 | 271 | 298 | 130 | 134 | 85 | 85 | 85 | 89 | 162 | 166 | 64 | 64 | 177 | 177 | 206 | 208 | 168 | 178 |
| 2ndPPs_28 | 2023seedlings | 90 | 94 | 219 | 222 | 154 | 160 | 268 | 271 | 130 | 130 | 79 | 79 | 85 | 89 | 162 | 166 | 64 | 66 | 173 | 173 | 206 | 208 | 178 | 178 |
| 2ndPPs_29 | 2023seedlings | 68 | 90 | 219 | 222 | 154 | 154 | 268 | 271 | 130 | 130 | 85 | 85 | 85 | 89 | 166 | 170 | 64 | 66 | 177 | 177 | 206 | 208 | 168 | 178 |
| 2ndPPs_30 | 2023seedlings | 90 | 94 | 219 | 222 | 154 | 160 | 268 | 271 | 130 | 130 | 79 | 85 | 89 | 89 | 162 | 170 | 64 | 66 | 173 | 173 | 206 | 208 | 168 | 178 |
| PB035 | PB | 74 | 90 | 236 | 238 | 158 | 158 | 286 | 286 | 130 | 130 | 79 | 85 | 81 | 81 | 162 | 162 | 60 | 64 | 161 | 161 | 204 | 206 | 174 | 176 |
| PB034 | PB | 74 | 74 | 236 | 238 | 158 | 160 | 286 | 286 | 130 | 130 | 79 | 85 | 81 | 81 | 162 | 162 | 60 | 64 | 163 | 163 | 204 | 204 | 174 | 176 |
| PB221114-0 | PB | 74 | 74 | 191 | 235 | 174 | 174 | 240 | 284 | 148 | 148 | 81 | 85 | 81 | 81 | 162 | 162 | 64 | 74 | 169 | 169 | 206 | 206 | 172 | 174 |
| PB045 | PB | 74 | 83 | 220 | 238 | -9 | -9 | 270 | 288 | 130 | 130 | 79 | 85 | 81 | 81 | 162 | 166 | 62 | 74 | 173 | 173 | 204 | 206 | 168 | 174 |
| PB044 | PB | 74 | 83 | 220 | 230 | 174 | 178 | 270 | 279 | 130 | 130 | 79 | 79 | 81 | 81 | 162 | 166 | 60 | 70 | 163 | 165 | 206 | 206 | 174 | 174 |
| PB022 | PB | 74 | 79 | 215 | 237 | 158 | 164 | 264 | 286 | 130 | 130 | 79 | 79 | 81 | 85 | 162 | 162 | 60 | 74 | 161 | 161 | 204 | 204 | 174 | 174 |
| PB059 | PB | 74 | 74 | 204 | 280 | 164 | 178 | 253 | 328 | 130 | 130 | 79 | 79 | 81 | 81 | 162 | 164 | 64 | 66 | 169 | 169 | 206 | 206 | 174 | 174 |
| PB019 | PB | 72 | 74 | 189 | 199 | 164 | 174 | 238 | 249 | 132 | 136 | 79 | 81 | 81 | 87 | 162 | 162 | 66 | 74 | 165 | 165 | 204 | 208 | 172 | 172 |
| PB020 | PB | 79 | 83 | 204 | 288 | 158 | 178 | 253 | 337 | 130 | 130 | 79 | 85 | 81 | 81 | 162 | 164 | 68 | 68 | 163 | 165 | 206 | 208 | 172 | 178 |
| PB001 | PB | 74 | 74 | 191 | 216 | 156 | 166 | 240 | 266 | 130 | 134 | 85 | 85 | 71 | 81 | 162 | 162 | 64 | 74 | 169 | 169 | 208 | 208 | 172 | 172 |
| PB221120-1 | PB | 74 | 83 | 192 | 216 | 160 | 166 | 242 | 266 | 130 | 130 | 81 | 85 | 81 | 81 | 162 | 162 | 60 | 64 | 173 | 173 | 204 | 206 | 174 | 174 |
| PB221120-2 | PB | 74 | 74 | 280 | 320 | 158 | 166 | 328 | 328 | 130 | 142 | 79 | 79 | 81 | 81 | 162 | 164 | 64 | 72 | 169 | 175 | 204 | 206 | 168 | 174 |
| PB221120-3 | PB | 74 | 79 | 285 | 285 | 158 | 170 | 334 | 334 | 130 | 130 | 79 | 81 | 81 | 81 | 162 | 162 | 64 | 68 | 169 | 169 | 206 | 206 | 174 | 174 |
| PB221120-4 | PB | 74 | 79 | 246 | 282 | 164 | 164 | 295 | 331 | 130 | 130 | 79 | 79 | 81 | 81 | 162 | 164 | 60 | 62 | 169 | 169 | 204 | 206 | 174 | 174 |
| PB023 | PB | 79 | 83 | 251 | 255 | 166 | 166 | 300 | 304 | 130 | 130 | 79 | 81 | 81 | 81 | 162 | 162 | 60 | 64 | 173 | 173 | 206 | 206 | 174 | 174 |
| PB221113-1 | PB | 74 | 74 | 191 | 235 | 174 | 174 | 240 | 284 | 148 | 148 | 80 | 84 | 81 | 81 | 162 | 162 | 64 | 74 | 165 | 169 | 206 | 206 | 174 | 176 |
| PB_PC221114-1 | PB | 70 | 83 | 248 | 248 | -9 | -9 | 298 | 340 | 130 | 130 | 79 | 79 | 81 | 81 | 162 | 164 | 64 | 64 | 163 | 165 | 206 | 208 | 168 | 174 |
| PC007 | PC | 72 | 90 | 191 | 196 | 178 | 178 | 240 | 245 | 130 | 150 | 79 | 81 | 81 | 81 | 162 | 164 | 68 | 80 | 171 | 171 | 206 | 206 | 174 | 178 |
| PC001 | PC | 81 | 83 | 189 | 220 | 158 | 158 | 238 | 270 | 138 | 158 | 79 | 79 | 81 | 81 | 164 | 164 | 58 | 68 | 163 | 163 | 206 | 206 | 174 | 174 |
| PC021 | PC | 83 | 96 | 194 | 197 | 168 | 168 | 242 | 247 | 150 | 156 | 79 | 79 | 81 | 81 | 164 | 164 | 70 | 74 | 177 | 177 | 204 | 206 | 174 | 174 |
| PC020 | PC | 72 | 81 | 176 | 208 | 158 | 178 | 226 | 257 | 128 | 148 | 79 | 85 | 81 | 81 | 162 | 164 | 70 | 70 | 163 | 163 | 208 | 208 | 172 | 178 |
| PC014 | PC | 70 | 86 | 197 | 226 | 160 | 160 | 246 | 275 | 138 | 138 | 79 | 79 | 81 | 83 | 164 | 168 | 54 | 70 | 163 | 163 | 206 | 212 | 174 | 174 |
| PC028 | PC | 72 | 72 | 246 | 246 | 160 | 176 | 295 | 295 | 128 | 138 | 79 | 79 | 81 | 81 | 164 | 166 | 68 | 70 | 163 | 163 | 208 | 208 | -9 | -9 |
| PC024 | PC | 83 | 98 | 187 | 221 | 151 | 156 | 236 | 270 | 130 | 130 | 79 | 79 | 81 | 81 | 164 | 164 | 68 | 80 | 177 | 177 | 206 | 206 | 174 | 178 |
| PC246 | PC | 72 | 79 | 191 | 207 | 170 | 178 | 240 | 257 | 158 | 158 | 79 | 79 | 80 | 80 | 164 | 164 | 70 | 74 | 163 | 163 | 206 | 210 | 168 | 174 |
| PC247 | PC | 72 | 104 | 209 | 214 | 158 | 158 | 258 | 264 | 130 | 140 | 79 | 79 | 80 | 80 | 162 | 164 | 66 | 70 | 163 | 163 | 206 | 206 | 174 | 174 |
| PC221113-1 | PC | 83 | 83 | 222 | 222 | 178 | 178 | 272 | 272 | 158 | 166 | 79 | 79 | 81 | 81 | 164 | 164 | 72 | 72 | 169 | 169 | 208 | 210 | 174 | 174 |
| PC221114-2 | PC | 72 | 94 | 209 | 212 | 158 | 158 | 258 | 262 | 138 | 154 | 79 | 79 | 81 | 81 | 164 | 164 | 66 | 74 | 163 | 173 | 206 | 206 | 174 | 174 |
| PCk22-2 | PC | 70 | 92 | 215 | 215 | 151 | 166 | 264 | 264 | 128 | 138 | 79 | 79 | 80 | 80 | 164 | 164 | 72 | 74 | 163 | 163 | 206 | 208 | 168 | 174 |
| PC221119-1 | PC | 72 | 72 | 201 | 208 | 156 | 156 | 251 | 257 | 128 | 150 | 79 | 79 | 65 | 81 | 162 | 164 | 60 | 70 | 163 | 167 | 206 | 206 | 168 | 174 |
| PC221119-0 | PC | 81 | 83 | 197 | 197 | 158 | 178 | 247 | 247 | 128 | 142 | 85 | 89 | 81 | 81 | 164 | 164 | 70 | 74 | 163 | 163 | 206 | 206 | 172 | 172 |
| PC221127-1 | PC | 72 | 86 | 226 | 226 | 160 | 160 | 275 | 275 | 138 | 150 | 79 | 79 | 80 | 82 | 164 | 164 | 70 | 72 | 163 | 163 | 206 | 206 | 174 | 174 |
| 13unk1 | PH | 72 | 72 | 212 | 212 | 154 | 164 | 262 | 262 | 130 | 134 | 79 | 85 | 67 | 67 | 172 | 172 | 66 | 66 | 171 | 171 | 208 | 208 | 174 | 174 |
| 14unk2 | PH | 72 | 72 | 182 | 212 | 139 | 139 | 232 | 262 | 130 | 134 | 79 | 87 | 67 | 67 | 172 | 172 | -9 | -9 | 173 | 173 | 208 | 208 | 174 | 174 |
| 15unk3 | PH | 72 | 72 | 182 | 212 | 139 | 139 | 232 | 262 | 130 | 134 | 79 | 87 | 67 | 67 | 172 | 172 | 64 | 66 | 173 | 173 | 198 | 200 | 174 | 174 |
| 17omt2 | PH | 72 | 72 | 182 | 214 | 139 | 164 | 232 | 262 | 132 | 132 | 79 | 85 | 79 | 79 | -9 | -9 | 66 | 66 | 171 | 171 | 208 | 224 | 174 | 174 |
| 18omt3 | PH | 72 | 72 | 182 | 207 | 139 | 164 | 232 | 256 | 132 | 132 | 79 | 87 | 67 | 67 | 174 | 174 | 64 | 66 | 173 | 177 | 208 | 224 | 174 | 174 |
| 19omt4 | PH | 72 | 72 | 182 | 214 | 154 | 154 | 232 | 262 | 132 | 132 | 85 | 87 | 67 | 67 | 174 | 176 | 66 | 66 | 170 | 170 | 224 | 224 | 174 | 174 |
| 21omt6 | PH | 72 | 72 | 212 | 214 | 139 | 164 | 262 | 262 | 132 | 132 | 79 | 79 | 67 | 67 | 174 | 180 | 66 | 66 | 173 | 173 | 208 | 208 | 168 | 180 |
| 22omt7 | PH | 72 | 72 | 207 | 212 | 139 | 164 | 256 | 262 | 132 | 132 | 85 | 87 | 67 | 67 | 162 | 174 | 64 | 66 | 173 | 173 | 224 | 224 | 174 | 174 |
| 23omt7 | PH | -9 | -9 | 212 | 214 | 154 | 154 | 262 | 262 | 132 | 132 | 79 | 79 | 69 | 69 | 174 | 184 | 66 | 66 | 171 | 173 | 198 | 198 | 174 | 174 |
